# Reassessing Reported Deaths and Estimated Infection Attack Rate during the First 6 Months of the COVID-19 Epidemic, Delhi, India

**DOI:** 10.3201/eid2804.210879

**Published:** 2022-04

**Authors:** Margarita Pons-Salort, Jacob John, Oliver J. Watson, Nicholas F. Brazeau, Robert Verity, Gagandeep Kang, Nicholas C. Grassly

**Affiliations:** Imperial College London School of Public Health, London, UK (M. Pons-Salort, O.J. Watson, N.F. Brazeau, R. Verity, N.C. Grassly);; Christian Medical College, Vellore, India (J. John, G. Kang)

**Keywords:** COVID-19, coronavirus disease, severe acute respiratory syndrome coronavirus 2, SARS-CoV-2, infection attack rate, infection fatality ratio, epidemics, mathematical modeling, respiratory infections, viruses, India, zoonoses

## Abstract

India reported >10 million coronavirus disease (COVID-19) cases and 149,000 deaths in 2020. To reassess reported deaths and estimate incidence rates during the first 6 months of the epidemic, we used a severe acute respiratory syndrome coronavirus 2 transmission model fit to data from 3 serosurveys in Delhi and time-series documentation of reported deaths. We estimated 48.7% (95% credible interval 22.1%–76.8%) cumulative infection in the population through the end of September 2020. Using an age-adjusted overall infection fatality ratio based on age-specific estimates from mostly high-income countries, we estimated that just 15.0% (95% credible interval 9.3%–34.0%) of COVID-19 deaths had been reported, indicating either substantial underreporting or lower age-specific infection-fatality ratios in India than in high-income countries. Despite the estimated high attack rate, additional epidemic waves occurred in late 2020 and April–May 2021. Future dynamics will depend on the duration of natural and vaccine-induced immunity and their effectiveness against new variants.

India had just under 150,000 reported coronavirus disease (COVID-19) deaths in 2020, fewer per 1 million persons than many other countries, such as Spain, France, the United Kingdom, and the United States (https://www.ourworldindata.org). This discrepancy could in part be because of a younger population but also because of incomplete documentation of overall deaths and of deaths with COVID-19 as a cause (*1*,*2*). Assessing the extent of underreporting of COVID-19 cases and deaths is essential for estimating actual disease burden and likely future trends in transmission.

Multiple severe acute respiratory syndrome coronavirus 2 (SARS-CoV-2) seroprevalence surveys conducted during 2020 in Delhi, one of India’s largest metropolitan areas (20 million residents), offered us an opportunity to assess the completeness of reported COVID-19 deaths and estimate the actual infection attack rate. SARS-CoV-2 transmission in Delhi has led to several waves of infection and death (Figure 1). At the beginning of the epidemic, all SARS-CoV-2 testing relied on reverse transcription PCR (RT-PCR), but after mid-June 2020, use of antigen-based rapid diagnostic tests (Ag-RDTs), which have lower sensitivity, quickly exceeded use of RT-PCR tests (Appendix Figure 1). Three serosurveys conducted in Delhi during 2020 that sampled participants >4 years of age found age- and sex-adjusted seropositivity rates (uncorrected for test sensitivity and specificity) of 22.8% in July, 28.7% in August, and 25.1% in September (Appendix Table 1) (*3*). The July survey found a difference in seropositivity between residents living inside or outside of slum areas (25.3% vs. 19.2%; p<0.001), but the August survey did not (28.9% vs. 28.8%; p = 0.94), and the September survey did not report this information.

**Figure 1:**
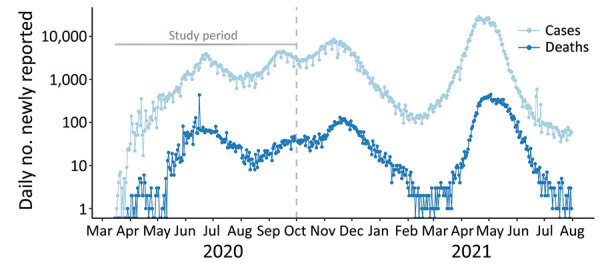
Daily number of coronavirus disease reported cases (light blue) and deaths (dark blue) on a logarithmic scale, Delhi, India, March 15, 2020–August 31, 2021. The gray vertical dashed line indicates the end of the study period, September 30, 2020.

## Materials and Methods

### Study Overview

**Figure 2:**
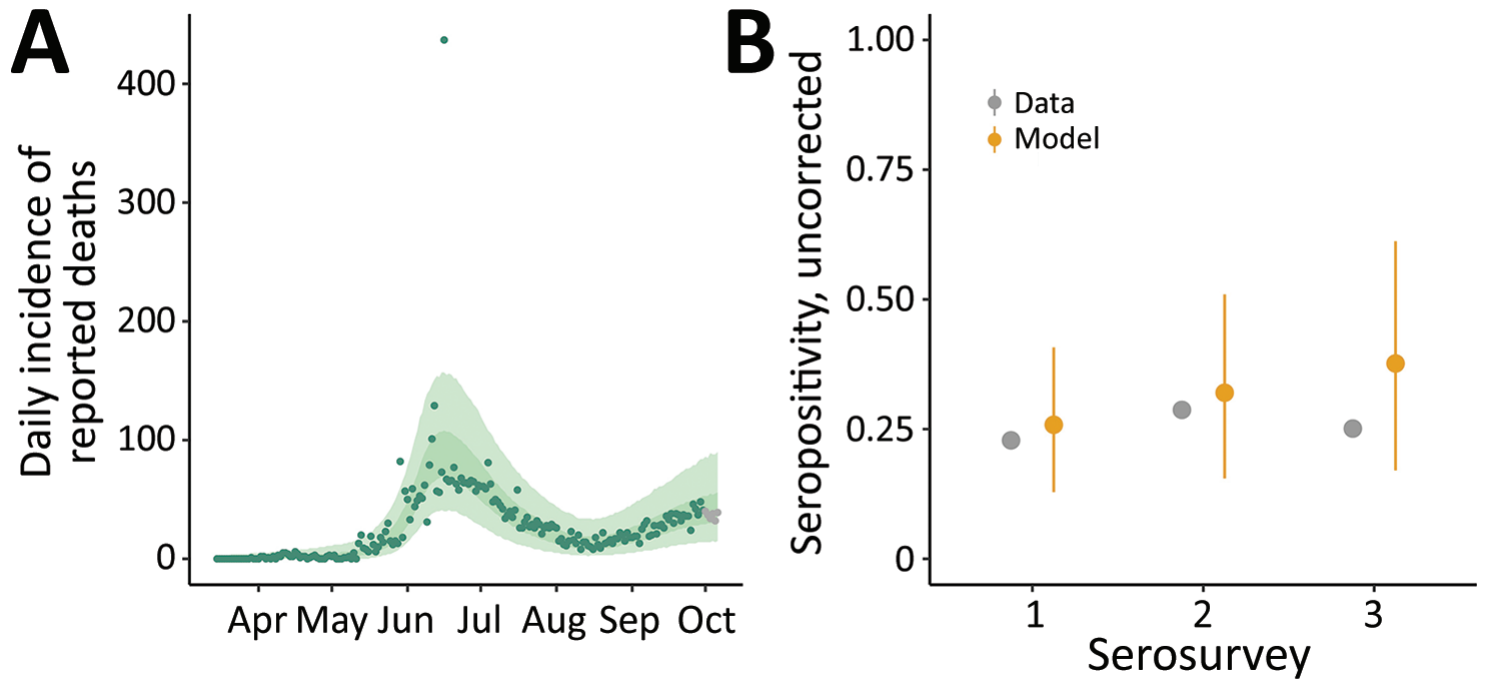
Model fit for reported coronavirus disease deaths and severe acute respiratory syndrome coronavirus 2 seroprevalence, Delhi, India, March 15–September 30, 2020. A) Model fit to the time-series of reported deaths (dots), showing 50% (darker green shading) and 95% (lighter green shading) credible intervals (CrI). The last 6 points (shown in gray) were not used for parameter inference. B) Model fit (orange dots) to seroprevalence data (gray dots) from 3 serosurveys, conducted in July, August, and September 2020, showing medians and 95% CrIs (error bars).

We developed a SARS-CoV-2 transmission model to estimate the incidence of infection and changes in the reproduction number (*R*) after the start of nonpharmaceutical interventions, including lockdowns (Appendix Table 2, Figure 2). We used Bayesian Markov chain Monte Carlo to fit the model to the 3 seroprevalence surveys and the time-series of reported deaths. We estimated the proportion of COVID-19 deaths reported by comparing reported deaths to the number expected based on the age-adjusted infection-fatality ratio (IFR) we used in the model. We used age-specific IFR estimates based on data from 7 countries in Europe; New York, USA; and Brazil (*4*) to estimate a median age-adjusted 0.39% IFR (95% prediction interval 0.21%–0.85%) for Delhi; median age-adjusted IFR in high-income countries with older populations, such as the United Kingdom, was ≈1%, based on data through June 2020 (*5*,*6*). The age-adjusted IFR for Delhi that we used was very similar to the 0.39% obtained using early data from China (*6*) and 0.40% from a meta-analysis based on data from advanced economies (as defined by membership in the Organization for Economic Cooperation and Development [https://www.oecd.org]) (*7*).

### Epidemiologic and Demographic Data

We obtained data on the number of confirmed SARS-CoV-2 cases and deaths reported daily in Delhi beginning March 14, 2020, from COVID19India (*8*), a volunteer-driven, crowdsourced initiative that collates data from several sources, including the Ministry of Health and Family Welfare. Cases and deaths that occurred before March 14 were reported as cumulative numbers. Because we did not know specifically when these pre–March 14 cases and deaths occurred, we did not use these data for parameter inference. For our model, we used data from the 3 serosurveys conducted in Delhi (*3*) on dates of sample collection, number of samples tested, seropositivity rate found, and reported estimates of sensitivity and specificity of the assay used in each of the three serosurveys (Appendix Table 1). We used projections of the 2021 population in Delhi from the National Commission on Population (*9*) and stratified the population by 10-year age groups.

### Transmission Model

To model SARS-CoV-2 transmission, we used a susceptible-exposed-infected-recovered (SEIR) deterministic transmission model (Appendix Figure 4).We did not stratify the population by age for the transmission parameters, assuming random mixing by age, meaning that epidemic growth was equivalent in all age groups in the model. We did not account for births or deaths from causes other than COVID-19 because of the model’s short timeframe.

Because epidemic growth rate is determined by the reproductive number and the generation time, *T_c_* (i.e., time interval between infection times of an infector-infectee pair) (*10*), we fixed the generation time to *T_c_* = 6.5 days on the basis of previous observations (*11*) and estimated the reproduction number. We split the generation time into the mean durations of the preinfectious (*d_E_* = 1/ω) and infectious (*d_I_* = 1/γ) periods, so that *T_c_ = d_E_ + d_I_* (*10*); we fixed *d_E_* and *d_I_* using information on the duration of the incubation period (i.e., time between infection and onset of symptoms) and the fact that infectiousness starts ≈1 day before symptoms start (*12*–*14*). Given an ≈5.5-day incubation (i.e., presymptomatic) period (*15*,*16*), to give the correct generation time, we assumed a mean duration of the preinfectious period of *d_E_* = 5.5 – 1.0 = 4.5 days and a mean duration of the infectious period of *d_I_* = 6.5 – 4.5 = 2 days.

### Disease Progression and Death Model

We modeled disease progression and death after infection independent of the transmission process (Appendix Figure 4). Because the model has been used for other purposes, it also included transitions to hospitalizations, but these were not relevant for our work and did not affect the results. We used the 5.5-day mean incubation period and a peaked distribution modeled with an Erlang distribution with shape parameter 6 (*15*). We assumed that one third of infections were asymptomatic, although there is high variability in the observed proportion of asymptomatic infections across studies (*17*–*19*).

We separately tracked the proportion of total infections leading to hospitalization and those leading to death; those hospitalized who eventually died were represented in both groups. We age-adjusted the proportion of infections leading to hospitalization with versus without critical care using demographics from Delhi and age-stratified estimates from China (*6*). That is, we computed a weighted average of the age-stratified estimates, assigning weights by the share of the corresponding age classes. We based the proportion of infections leading to death on estimates of age-stratified IFR (*4*) applied to the population of Delhi.

We set average time from symptom onset to hospitalization as 5.8 days, consistent with observations in China (*20*). For hospitalization without critical care, we assumed a mean 9.8-day stay; if critical care was required, we assumed 9.8 days in critical care, followed by 3.3 days recovery outside of critical care, based on early estimates from the United Kingdom (*21*). The average time from symptom onset to death was ≈16 days (*6*). Using these estimates, we assumed a 10-day mean for time between hospitalization and death. These values might differ for India, but no domestic data were available at that time.

### Parameter Inference

We fitted the transmission model to both the seroprevalence data and reported daily COVID-19 deaths (Appendix). We allowed the reproduction number to change at 5 different time points corresponding to changes in interventions (Appendix Table 2). Denoting the basic reproduction number during the first infection period (i.e., before any changes) as R_0_, the reproduction number after *i* number of changes as R*_i_* (*i* in periods 1–5), we conducted parameterization of R*_i_* as R*_i_* = R_0_ × (1 + *r_1_*) ×...× (1 + *r_i_*), where *r_1_*,…,*r_5_* measured the relative change in the reproduction number from one period to the previous one.

We estimated R_0_ and the subsequent changes at each time point, *r_1_*_,_…,*r_5_*, the initial number of infected [*E*(0) + *I*(0)], the reporting, θ, and overdispersion of deaths, *k*. We assumed February 19, 2020 (28 days before the first 10 cases were reported), as the starting time (*t*_0_) for the simulations and estimated the number of infected persons at that time point, [*E*(0) + *I*(0)]. To prevent parameter estimates being biased by the earliest phase of the epidemic, when underreporting of deaths might have been greatest, we computed likelihood using data collected from March 29, 2020, when the first COVID-19 death was reported, through September 30, 2020, the end of the 6-month study period.

We could not estimate a change in transmission at the first time point (*r*_1_), corresponding to the start of the lockdown on March 25, because no deaths were reported during March 15–28; we therefore assumed *r*_1_ = 0. We assumed May 4, when the first lockdown relaxations were introduced, as the next time point for change in the reproduction number (*r*_2_). Therefore, estimates of the reproduction number during February 19–May 4, 2020, from the beginning of the simulations through *r*_2_, implicitly accounted for any effects of the lockdown during that time. Because R_0_ was highly correlated with the initial number of infected, we estimated the total number of infections just before *r*_2_ and back-calculated the initial number of infected persons using a simple exponential growth model to define the relationship between R_0_ and the epidemic growth rate for a SEIR model (*9*,*22*). We performed 100,000 iterations using Markov chain Monte Carlo in the lazymcmc software package (*23*) and uniform prior distributions to estimate model parameters; we ran 4 chains with different starting values to check convergence. We performed all analyses using R version 4.0.2 (*24*).

## Results

**Figure 3:**
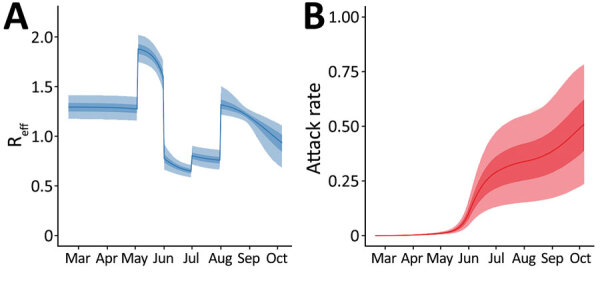
Effective reproduction number, R_eff_, for model of coronavirus disease deaths and severe acute respiratory syndrome coronavirus 2 attack rate, Delhi, India, March 15–September 30, 2020. A) Median and 50% (dark blue shading) and 95% (light blue shading) credible intervals (CrIs) of the estimated R_eff_ from the model. Changes were assumed to occur beginning when changes in the interventions were introduced. B) Median and 50% (dark red shading) and 95% (light red shading) CrIs of the estimated infection attack rate from the model.

Our model fit the data well for both the death time-series (Figure 2, panel A) and seroprevalence survey data (Figure 2, panel B), except for the last serosurvey, in which we estimated an increase in seropositivity from the previous survey, instead of a slight decrease. This difference might have been because the observation model did not account for waning antibodies and the possibility of seroreversion. However, the third serosurvey used a different testing kit, which might also have contributed to this difference. We estimated that the first peak in infection incidence was reached on May 31, at a median of 294,930 (95% credible interval [CrI] 143,271–440,702) new infections per day (Appendix Figure 5). Incidence at the second peak, reached on September 17, was lower, at a median of 79,032 (95% CrI 40,484–109,140) new infections per day. Assuming that changes in transmission occurred beginning at the times of each change in interventions and accounting for the reduction in susceptible persons, we estimated that the effective reproduction number, R_eff_, increased with the first relaxation of the lockdown introduced May 4 (beginning of phase 3); in June and July, during the first 2 reopening phases, R_eff_ was <1; in August, R_eff_ then increased again to >1 (Figure 3, panel A), resulting in a median infection attack rate of 48.7% (95% CrI 22.1%–76.8%) by the end of September. After that, Delhi experienced a large third wave of cases and deaths (Figure 1), suggesting that even with approximately half the population having been infected, the herd immunity threshold had not yet been reached at that time. Of interest, a serosurvey conducted in January 2021 found a sex- and age-adjusted seroprevalence of 56.1%, probably indicating a steep increase in the cumulative number of infections, reflecting the effects of this third wave of transmission.

**Figure 4:**
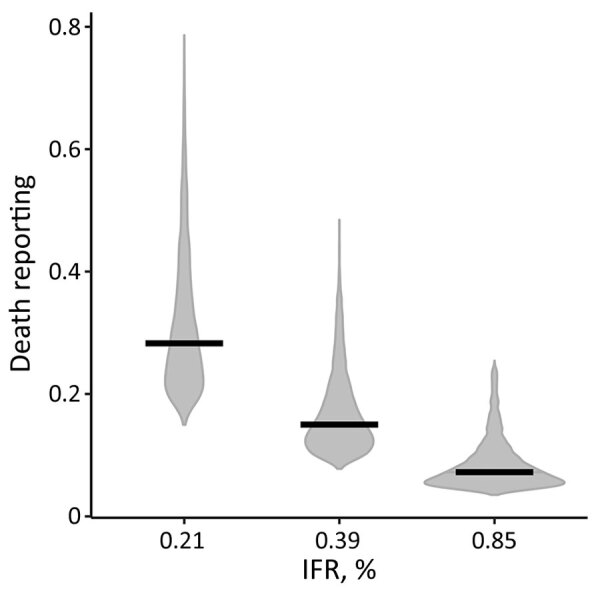
Estimated reporting of coronavirus disease deaths, Delhi, India, March 15–September 30, 2020. Violin plots show the posterior distribution of the estimate of death reporting for 3 different values for the assumed age-adjusted IFR, using age-stratified estimates of IFR based on data from mostly high-income countries; 0.21% corresponds to lower bound, 0.39% to the median, and 0.85% to the upper bounds of the IFR based on data documented elsewhere (*4*). Horizontal black lines indicate the median values of the posterior distributions. IFR, infection-fatality ratio.

Using a 0.39% age-adjusted IFR, we estimated reported deaths to be 15.0% (95% CrI 9.3%–34.0%) of actual deaths (Figure 4; Appendix Figure 6). Repeating the analysis using an age-adjusted IFR of 0.21%, corresponding to the lower bound of the 95% prediction interval for IFR based mostly on age-specific high-income country (HIC) data (*4*), increased the proportion of reported deaths to 28% (95% CrI 18–59%) of actual deaths (Figure 4).

**Figure 5:**
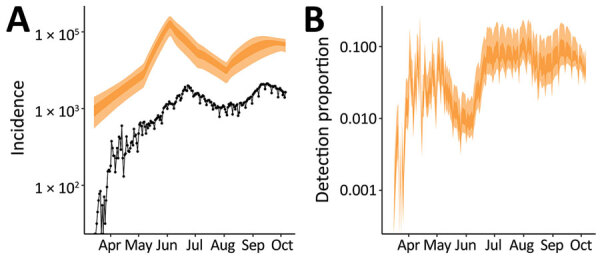
Reported cases of symptomatic severe acute respiratory syndrome coronavirus 2 infections and estimated actual number of cases, Delhi, India, March 15–September 30, 2020. A) Daily number of newly reported cases (black dots) and 50% (dark orange shading) and 95% (light orange shading) credible intervals (CrIs) for the estimated actual incidence of symptomatic infections, assuming that 2/3 infections are symptomatic. B) Estimated detection probability per symptomatic infection per day: 50% (dark orange) and 95% (light orange) CrIs.

On the basis of infection incidence determined using our model, we also estimated the probability of detecting COVID-19 cases over time by comparing the number of reported cases to the estimated incidence of symptomatic infections (Figure 5, panel A). The probability of detecting infection quickly increased over the last weeks of March, fluctuated until mid-June, then remained relatively consistent through the end of September; a median of 7.1% of all symptomatic infections was detected during July 1–September 30, 2020 (Figure 5, panel B).

## Discussion

The low proportion of reported deaths relative to actual deaths we found is consistent with findings from other cities in India, where seroprevalence surveys suggested substantially greater exposure to infection than predicted on the basis of reported COVID-19 deaths. For example, comparing seroprevalence during the first half of July 2020 in Mumbai (*25*) with cumulative deaths at that time suggested that only 21% of deaths were reported (Appendix Table 3). Similarly, a large-scale prospective, active-surveillance study conducted in the district of Madurai, Tamil Nadu, India, during the first wave of COVID-19 in summer 2020 found that only 11.0% of deaths were reported, compared with expected deaths based on IFR estimates from other settings (*26*). This high level of underreporting might reflect incomplete or delayed reporting of deaths and a failure to report COVID-19 as a suspected or confirmed cause of death, particularly in the absence of a SARS-CoV-2 test result.

The extent of underreporting might also reflect our use of an age-specific IFR for India derived from mostly HIC data. Age-specific IFR may be lower in India for several reasons. First, the prevalence of underlying medical conditions that increase the risk for severe COVID-19 after infection is somewhat lower in India than in the countries that informed the age-specific IFR estimates for our model (Appendix Figure 7) (*27*). However, correcting the Delhi IFR to account for the lower prevalence of underlying conditions only marginally reduced the age-adjusted IFR (<0.02%). Second, a recent study that analyzed COVID-19 deaths from Mumbai and Karnataka by age found that IFR rose less steeply with age than in HICs (R. Cai et al., unpub. data, https://doi.org/10.1101/2021.01.05.21249264). Third, differences in immunity reflecting exposure to a greater number of pathogens, including related coronaviruses, or simply lower frailty among those surviving to older ages in India compared with HICs could theoretically reduce the IFR in older groups, although data supporting these hypotheses are lacking (*28*; B. Chatterjee et al., unpub. data, https://doi.org/10.1101/2020.07.31.20165696). If the IFR in India was actually higher than in HICs, the proportion of deaths reported would be even lower. For example, using a 0.85% age-adjusted IFR, corresponding to the upper bound of the 95% prediction interval for IFR based on age-specific HIC data (*4*), would decrease the reported deaths to only 7% (95% CrI 4%–21%) of actual deaths (Figure 4).

The first limitation of our study is that we did not structure the transmission model by age, and therefore, did not account for differences in attack rates between age groups. However, age-structured models have predicted relatively homogeneous infection attack rates across age for India (*29*), consistent with age-stratified seroprevalence estimates (*3*), suggesting that any bias in our results from age-specific patterns of mixing and potentially lower attack rates in more susceptible older age groups is likely to be limited. Second, we assumed that the proportion of deaths reported was constant over the study period, but it might have changed over time. Therefore, our estimate of reported deaths represents an average over the study period. Finally, we used an age-specific IFR based on estimates mostly from HICs and explored sensitivity based on this assumption, including using data on underlying conditions in India. Further analyses using data from cohort studies or demographic surveillance specific to India will help to refine these estimates of IFR and the exact degree of underreporting of death.

The total number of new COVID-19 cases declined in India between mid-September 2020 and mid-February 2021 but started increasing again after that, and in April–May 2021, India experienced a devastating nationwide second epidemic wave bigger than the first one. How much of the country’s population had already been infected before the second nationwide wave and whether the herd immunity threshold had been reached were unclear (*30*). Seroprevalence surveys conducted in major cities, such as Mumbai, reported seroprevalence rates >50% in slum areas for the first half of July 2020 (*25*), suggesting that infection spread very quickly over the first few months of the epidemic in certain pockets. However, seroprevalence rates <20% in non–slum areas showed that the epidemic was spatially highly heterogeneous. Understanding what brought the number of cases down after the first wave in different parts of India and how to interpret the serosurvey results related to building population immunity are key to understanding and predicting the dynamics of subsequent waves of COVID-19.

The SARS-CoV-2 Delta variant emerged in Maharashtra in late 2020 and spread across India during the first few months of 2021, replacing other variants. In vitro data characterizing the Delta variant found that it was less sensitive to serum neutralizing antibodies from persons previously infected with other variants and that it also had higher replication efficiency (*31*). These findings suggest that the predominance of the Delta variant in the upsurge of SARS-CoV-2 cases seen in India during April and May 2021 resulted from either immune escape in previously infected persons, increased transmissibility, or both. These mechanisms, together with possible waning of population immunity over time, likely explain the increase in SARS-CoV-2 cases in Delhi, despite the high attack rate that we estimated in September 2020 and the high reported seroprevalence (≈56% for both) in the round 5 (January 2021) and 6 (April 2021) cross-sectional serosurveys. Analysis of epidemiologic data is needed to disentangle how these mechanisms contributed to the second nationwide epidemic wave.

In conclusion, our analysis found reported COVID-19 deaths in Delhi during the first 6 months of the pandemic were well below the number of actual deaths. Our estimate of underreporting of deaths might reflect incomplete or delayed documentation or failure to report COVID-19 as a cause of death but may also reflect our use of an age-specific IFR, for India, derived from mostly HIC data.. 

Appendix Additional information on mathematical modeling of actual attack rates and deaths early in the coronavirus disease epidemic, Mumbai, India
