# Supplementary material for: Reassessing Reported Deaths and Estimated Infection Attack Rate during the First 6 Months of the COVID-19 Epidemic, Delhi, India
Source: Emerg Infect Dis. 2022 Apr;28(4):759–66. doi: 10.3201/eid2804.210879 (PMC8962916; doi:10.3201/eid2804.210879)
Supplement: Appendix — Additional information on mathematical modeling of actual attack rates and deaths early in the coronavirus disease epidemic, Mumbai, India [file 21-0879-Techapp-s1.pdf]

Article DOI: https://doi.org/10.3201/eid2804.210879

Reassessing Reported Deaths and Estimated Infection Attack Rate during the First 6 Months of the COVID-19 Epidemic, Delhi, India

Appendix

Parameter Inference: Observation Models

To account for underreporting and overdispersion of the death data, we modeled the number of deaths with a negative binomial distribution:

$$deaths^{obs}(t) \sim NegBin(\mu = \theta \times deaths(t), k)$$

where the mean is $\theta \times deaths(t)$ and the variance is $\theta \times deaths(t) + (\theta \times deaths(t))^2 / (\theta \times deaths(t))^k$.

We modeled the number of persons who would test seropositive each day with a given serological assay of sensitivity Se_j and specificity Sp_j as

$$seropositives_j(t) = \sum_{i=1}^{t-1} e_{inc}(i) * p_{sero,j}(t-i) + (1 - Sp_j) * s_{prev}(t)$$

where e_{inc} was the incidence of infection, $p_{sero,j}(\tau)$ was the probability of testing positive τ days after infection and s_{prev} was the number of susceptible persons, as in (J. Ojal, unpub. data). We assumed that $p_{sero,j}(\tau)$ increases linearly from day 0 of infection to Se_j 26 days after infection and remains constant after that (we did not consider seroreversion):

$$p_{sero,j}(\tau) = \begin{cases} Sej \times \frac{\tau}{26}, & \text{if } 0 < \tau < 26 \\ Sej, & \text{if } \tau \geq 26 \end{cases}$$

Finally, we linked the modelled number of seropositives at the mid–time point of each serosurvey, denoted here by ts_1 for survey 1, ts_2 for survey 2, and ts_3 for survey 3, to the data

from the 3 seroprevalence surveys using Beta binomial distributions to account for overdispersion:

$$seropositives(t_{sj})^{obs} \sim$$

$$BetaBinom(N = N_{samples,j}, p = {seropositives_j(t_{sj})}/{population}, \sigma = 1/0.7)$$

with the overdispersion parameter fixed to $\sigma = 1.0/0.7$, because of the small number of observations available to accurately estimate it.

Equations for the Transmission Model

See notation and description of variables in Appendix Table 4 and parameters in Appendix Table 5.

$$\frac{dS}{dt} = -\lambda S$$

$$\frac{dE}{dt} = \lambda S - \omega E$$

$$\frac{dI}{dt} = \omega E - \gamma I$$

$$\frac{dR}{dt} = \gamma I$$

$$\lambda = R_i \gamma \frac{I}{N}$$

$$N = S + E + I + R$$

Equations for the Disease-Progression Model

See notation and description of variables in Appendix Table 4 and parameters in Appendix Table 5.

$$\frac{dPSY_1}{dt} = \lambda S - \frac{6}{D_{PSY}} PSY_1$$

$$\frac{dPSY_2}{dt} = \frac{6}{D_{PSY}} PSY_1 - \frac{6}{D_{PSY}} PSY_2$$

…

$$\frac{dPSY_6}{dt} = \frac{6}{D_{PSY}} PSY_5 - \frac{6}{D_{PSY}} PSY_6$$

$$\frac{d(SY + ASY)}{dt} = \frac{6}{D_{PSY}} PSY_6 - \frac{1}{D_{SY}} (SY + ASY)$$

$$\frac{dHNC}{dt} = p_{inf,hnc} \frac{1}{D_{SY}} (SY + ASY) - \frac{1}{D_{HNC}} HNC$$

$$\frac{dHC}{dt} = p_{inf,hc} \frac{1}{D_{SY}} (SY + ASY) - \frac{1}{D_{HC}} HC$$

$$\frac{dHSD}{dt} = p_{hc,hsd} \frac{1}{D_{HC}} HC - \frac{1}{D_{HSD}} HSD$$

$$\frac{dF}{dt} = p_{inf,death} \frac{1}{D_{SY}} (SY + ASY) - \frac{1}{D_F} F$$

$$\frac{dD}{dt} = \frac{1}{D_F} F$$

References

1. Sharma N, Sharma P, Basu S, Saxena S, Chawla R, Dushyant K, et al. The seroprevalence of severe acute respiratory syndrome coronavirus 2 in Delhi, India: a repeated population-based seroepidemiological study. Trans R Soc Trop Med Hyg. 2021;trab109. PubMed https://doi.org/10.1093/trstmh/trab109

2. Malani A, Shah D, Kang G, Lobo GN, Shastri J, Mohanan M, et al. Seroprevalence of SARS-CoV-2 in slums versus non-slums in Mumbai, India. Lancet Glob Health. 2020;9:e110–1.

3. Bi Q, Wu Y, Mei S, Ye C, Zou X, Zhang Z, et al. Epidemiology and transmission of COVID-19 in 391 cases and 1286 of their close contacts in Shenzhen, China: a retrospective cohort study. Lancet Infect Dis. 2020;20:911–9. PubMed https://doi.org/10.1016/S1473-3099(20)30287-5

4. Lauer SA, Grantz KH, Bi Q, Jones FK, Zheng Q, Meredith HR, et al. The incubation period of coronavirus disease 2019 (COVID-19) from publicly reported confirmed cases: estimation and application. Ann Intern Med. 2020;172:577–82. PubMed https://doi.org/10.7326/M20-0504

5. Lau YC, Tsang TK, Kennedy-Shaffer L, Kahn R, Lau EHY, Chen D, et al. Joint estimation of generation time and incubation period for coronavirus disease (Covid-19). J Infect Dis. 2021;jiab424. PubMed https://doi.org/10.1093/infdis/jiab424

6. Nishiura H, Linton NM, Akhmetzhanov AR. Serial interval of novel coronavirus (COVID-19) infections. Int J Infect Dis. 2020;93:284–6. PubMed https://doi.org/10.1016/j.ijid.2020.02.060

7. Xin H, Li Y, Wu P, Li Z, Lau EHY, Qin Y, et al. Estimating the latent period of coronavirus disease 2019 (COVID-19). Clin Infect Dis. 2021;ciab746. PubMed https://doi.org/10.1093/cid/ciab746

8. Verity R, Okell LC, Dorigatti I, Winskill P, Whittaker C, Imai N, et al. Estimates of the severity of coronavirus disease 2019: a model-based analysis. Lancet Infect Dis. 2020;20:669–77. PubMed https://doi.org/10.1016/S1473-3099(20)30243-7

9. Clark A, Jit M, Warren-Gash C, Guthrie B, Wang HHX, Mercer SW, et al.; Centre for the Mathematical Modelling of Infectious Diseases COVID-19 working group. Global, regional, and national estimates of the population at increased risk of severe COVID-19 due to underlying health conditions in 2020: a modelling study. Lancet Glob Health. 2020;8:e1003–17. PubMed https://doi.org/10.1016/S2214-109X(20)30264-3

Appendix Table 1. Information on the 3 serosurveys conducted in Delhi (*1*) during the study period.*,†

Serosurvey no. (agency)	Midtime point	Samples, no.	Uncorrected seropositivity rate	Test sensitivity	Test specificity
1 (NCDC)	07/01/2020	19,041	0.2283	0.921	0.977
2 (MAMC)	08/04/2020	15,046	0.287	0.921	0.977
3 (MAMC)	09/04/2020	17,409	0.251	0.9912	0.9933

NCDC, National Center for Disease Control; MAMC: Maulana Azad Medical College
†Serosurveys 1 and 2 used the testing kit ELISA COVID-Kawach kit, serosurvey 3 used the ERBALISA COVID-19 IgG.

Appendix Table 2. National interventions to control COVID-19 in India.

Intervention	Start date	End date	Modeled change in R?	Observations
Janata curfew	03/22/2020	03/22/2020	No	14-hour curfew
Phase 1 lockdown	03/25/2020	04/14/2020	No*	Beginning of lockdown
Phase 2 lockdown	04/15/2020	05/03/2020	No	Lockdown extended
Phase 3 lockdown	05/04/2020	05/17/2020	Yes	Lockdown extended but with relaxations
Phase 4 lockdown	05/18/2020	05/31/2020	No	Lockdown extended
Unlock 1.0	06/01/2020	06/30/2020	Yes	Reopening phase
Unlock 2.0	07/01/2020	07/31/2020	Yes	Reopening phase
Unlock 3.0	08/01/2020	08/31/2020	Yes	Reopening phase
Unlock 4.0	09/01/2020	09/30/2020	No	Reopening phase

*Insufficient data available to estimate a change in transmission at that point.

Appendix Table 3. Estimate of death reporting in Delhi and Mumbai based on a simple back calculation based on data on the cumulative number of deaths up until the first serosurvey. For Mumbai, we used the seroprevalence data reported elsewhere (*2*), we assume that 53% of the population lives in slums, and account for different age distribution in slums and non-slums, based on data from the TIFR Covid-19 City-Scale Simulation Team (P. Harsha, unpub.data).

Area	Date†	Cumulative reported deaths	Seroprevalence, %‡	Population size, M	IFR, %¶	Deaths reported, %
Delhi	07/01/2020	2,803	22.86	20.86	0.39	15
Mumbai	07/07/2020	3,317	NA	12.8	NA	21
Slums	NA	NA	55.7	6.784	0.29	NA
Non-slums	NA	NA	16.2	6.016	0.51	NA

*IFR, infection fatality ratio; M, millions; NA, not available
†Midtime point of sample collection during the serosurvey.
‡Corrected for test sensitivity and specificity.
¶Based on model described elsewhere (N.F. Brazeau, unpub. data).

Appendix Table 4. Model variables.

Notation	Description
S	Susceptible
E	Exposed
I	Infected
R	Recovered
N	Total population size
PSY1…PSY6	Presymptomatic 1…6*
SY	Symptomatic
ASY	Asymptomatic
HNC	Hospitalized, no critical care
HC	Hospitalized, critical care
HSD	Hospitalized, stepdown
F	Fatal infections
D	Deaths

*The 6 compartments were necessary to model the Erlang distribution with shape value 6)

Appendix Table 5. Model parameters

Notation	Description	Value and units	Reference
R_0	Basic reproduction number	Estimated	NA
r1…r5	Coefficients modifying the reproduction number	Estimated	NA
1/ω	Mean duration preinfectious period	4.5 d	Set using information on the generation time (*3*), the duration of the pre-symptomatic period (*4*) and the fact that infectiousness starts about one day before symptoms start (*5*–*7*) (see Methods for details)
1/γ	Mean duration infectious period	2 d	As above
D_{PSY}	Mean duration presymptomatic	5.5 d	(*4*)
D_{SY}	Mean duration symptomatic	5.8 d	(K. Gaythorpe, unpub. data)
D_{HNC}	Mean duration hospitalized non–critical care	9.8 d	Based on early, unpublished data from the UK
D_{HC}	Mean duration hospitalized critical care	9.8 d	Based on early, unpublished data from the UK
D_{HSD}	Mean duration hospitalized step-down	3.3 d	Based on early, unpublished data from the UK.
D_F	Mean duration infections leading to fatal outcome	10 d	Based on a mean time from symptom onset to hospitalization of 5.8 days (K. Gaythorpe, unpub. data) and an average time from symptom onset to death of ≈16 days (*8*)
pINF,HNC	Proportion of infections leading to hospitalization for noncritical care (age-adjusted)	2.77×10^{-2}	(*8*)
pINF,HC	Proportion of infections leading to hospitalization for critical care (age-adjusted)	6.64×10^{-3}	(*8*)
pINF,D	Proportion of infections leading to death (age-adjusted IFR)	3.94×10^{-3} †	(N.F. Brazeau, unpub. data)
pHC,HSD	Proportion of hospitalizations in critical care recovering (in stepdown compartment)	0.6	Working assumption

***IFR, infection fatality ratio; NA, not applicable**
†Value used in the main analysis. Sensitivity analysis performed based on this value.

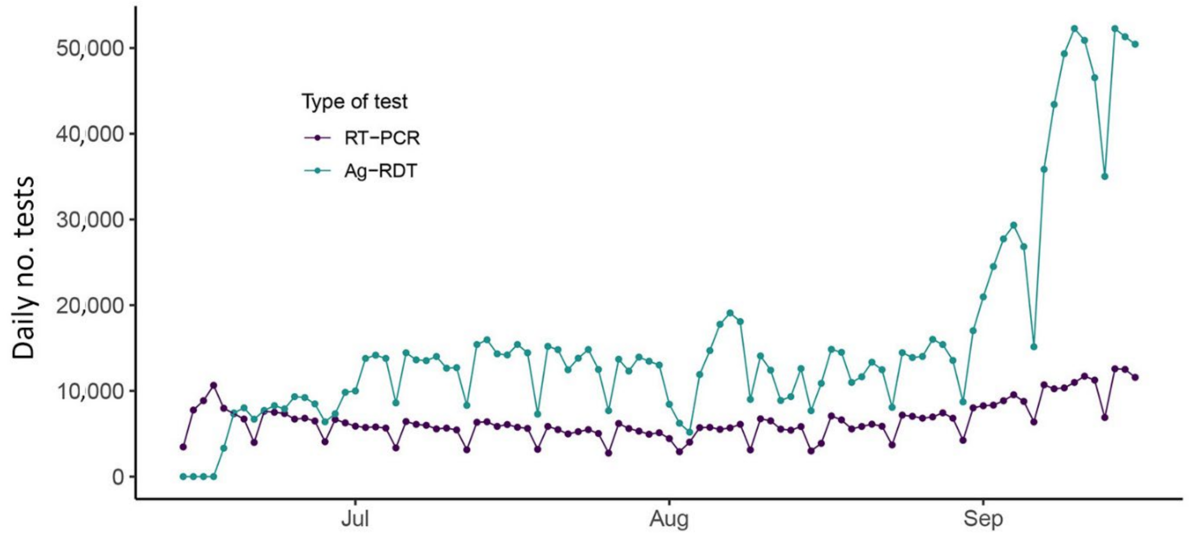

Appendix Figure 1. Daily number of reverse transcription PCR and antigen-detecting rapid diagnostic tests performed, June 14–September 16, 2020. Ag-RDT, antigen-detecting rapid diagnostic tests; RT-PCR, reverse transcription PCR

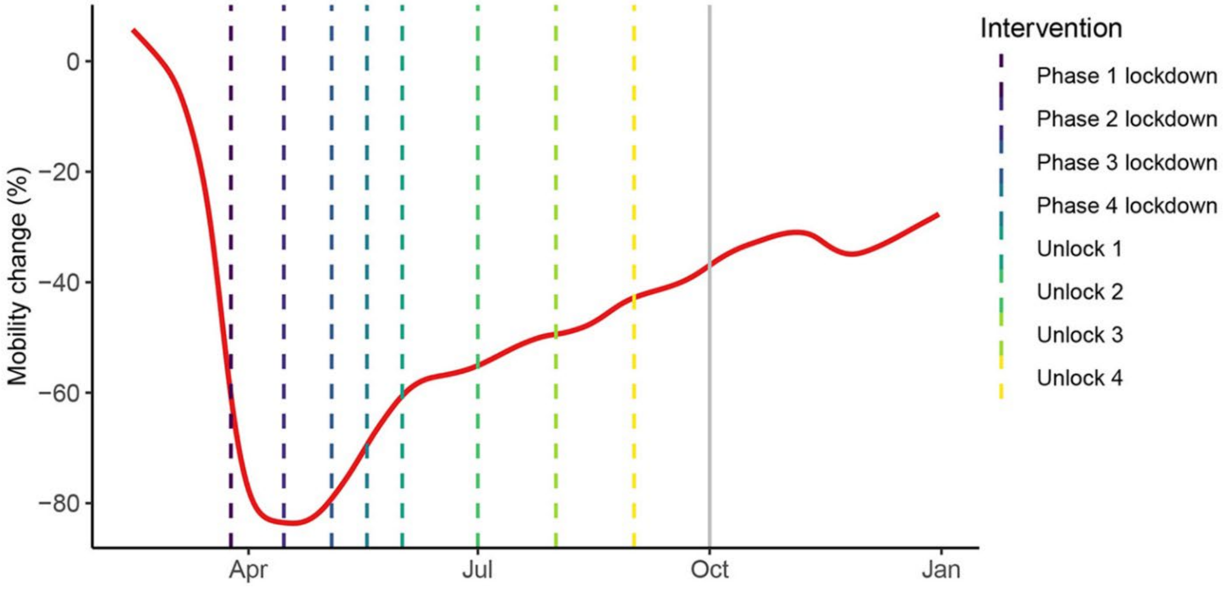

Appendix Figure 2. Changes in mobility over time (based on summarized Google data, February 15–December 31, 2020). The data were smoothed to remove weekend effect and averaged across 5 of the 6 data streams available (all except residential; Appendix Figure 3). Unlock 1–4 refers to gradual reopenings. Colored lines indicate the start of interventions (Appendix Table 2). Grey vertical line indicates the end of the study period (September 30, 2020).

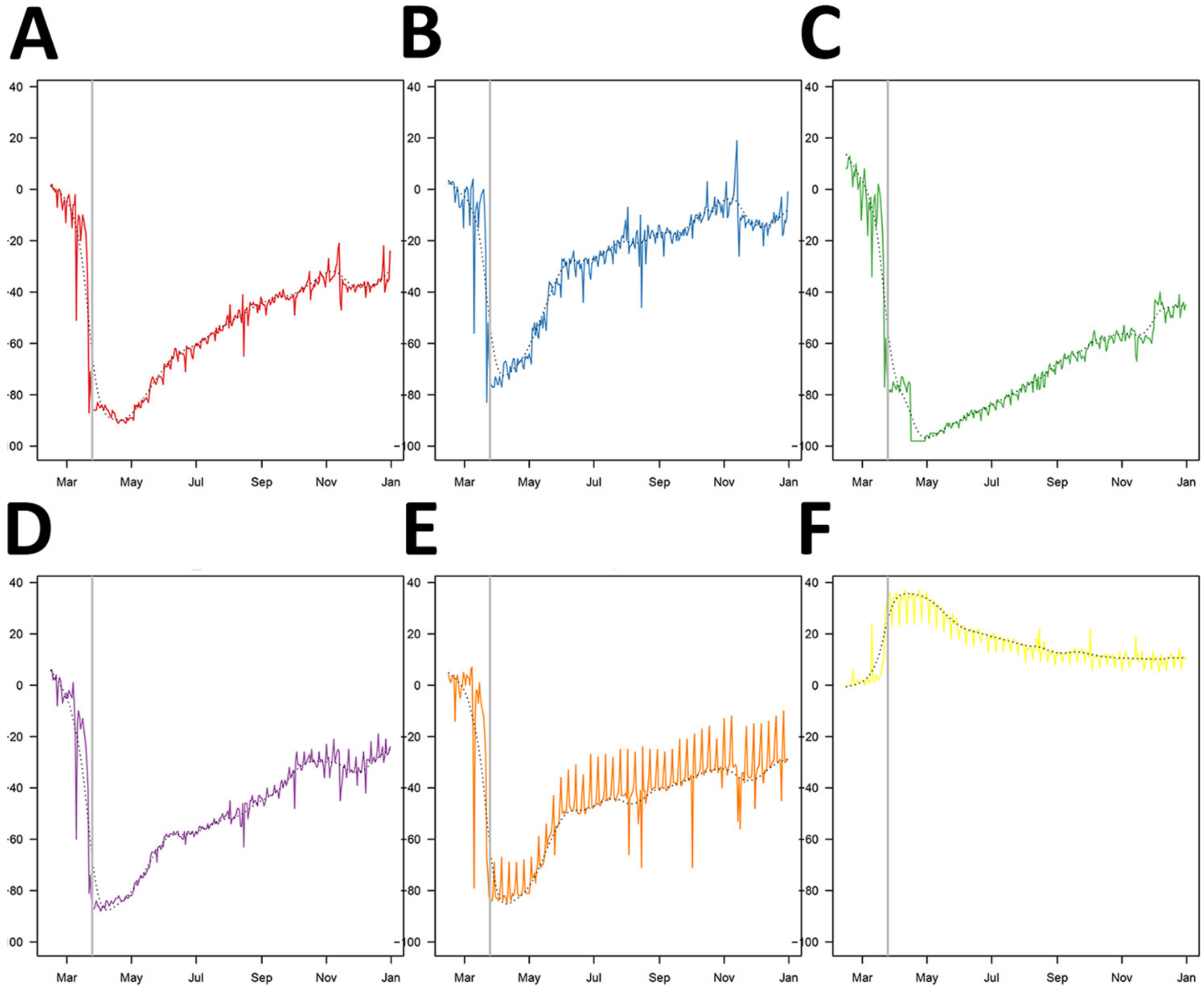

Appendix Figure 3. Changes in mobility over time for Delhi (based on raw data from Google). Raw data for the 6 available data streams are shown in red for retail and recreation (A), blue for grocery and pharmacy (B), green for parks (C), purple for transit stations (D), orange for workplaces (E), and in yellow for residential (F). Dashed black lines indicated smoothed raw data obtained with a spline after removing the points for Thursday to Sunday to remove the weekend effect. These smoothed lines for all the streams, except residential (F), were averaged to produce Appendix Figure 2. Vertical line in each panel indicates the beginning of the lockdown on March 25, 2020.

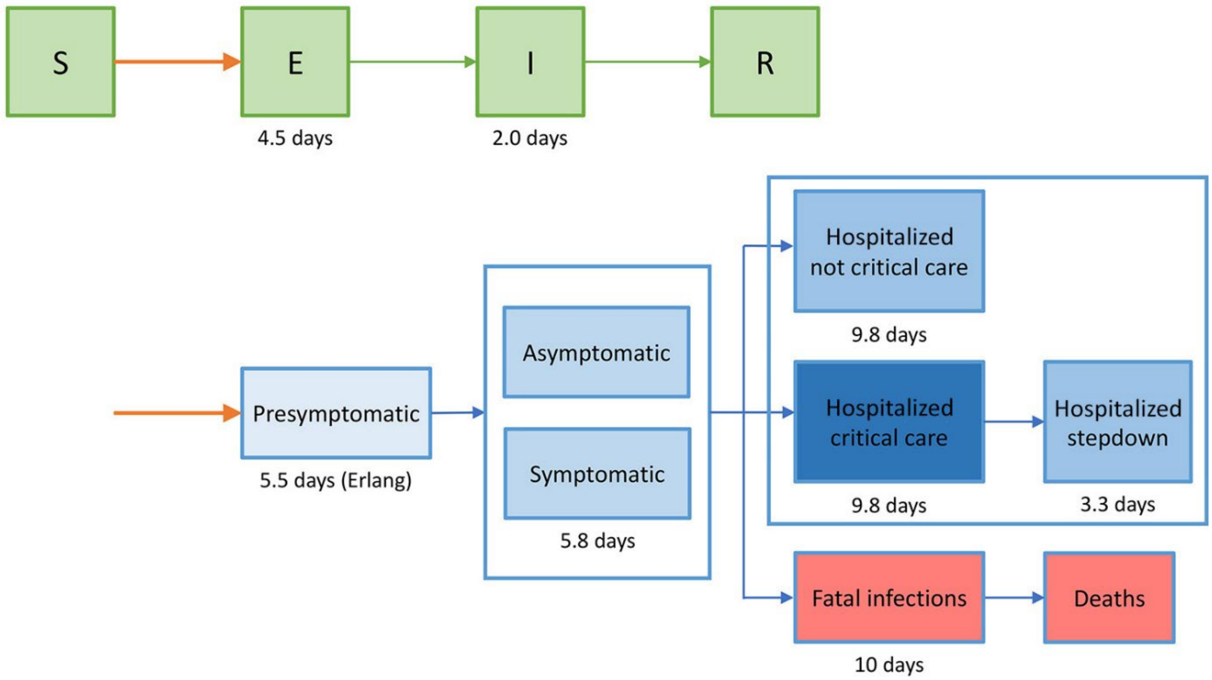

Appendix Figure 4. Model diagram. In green, the susceptible-exposed-infectious-removed (SEIR) transmission model. Blue boxes: the disease-progression model; red boxes: infections leading to death. S, susceptible; E, exposed; I, infectious; R, removed

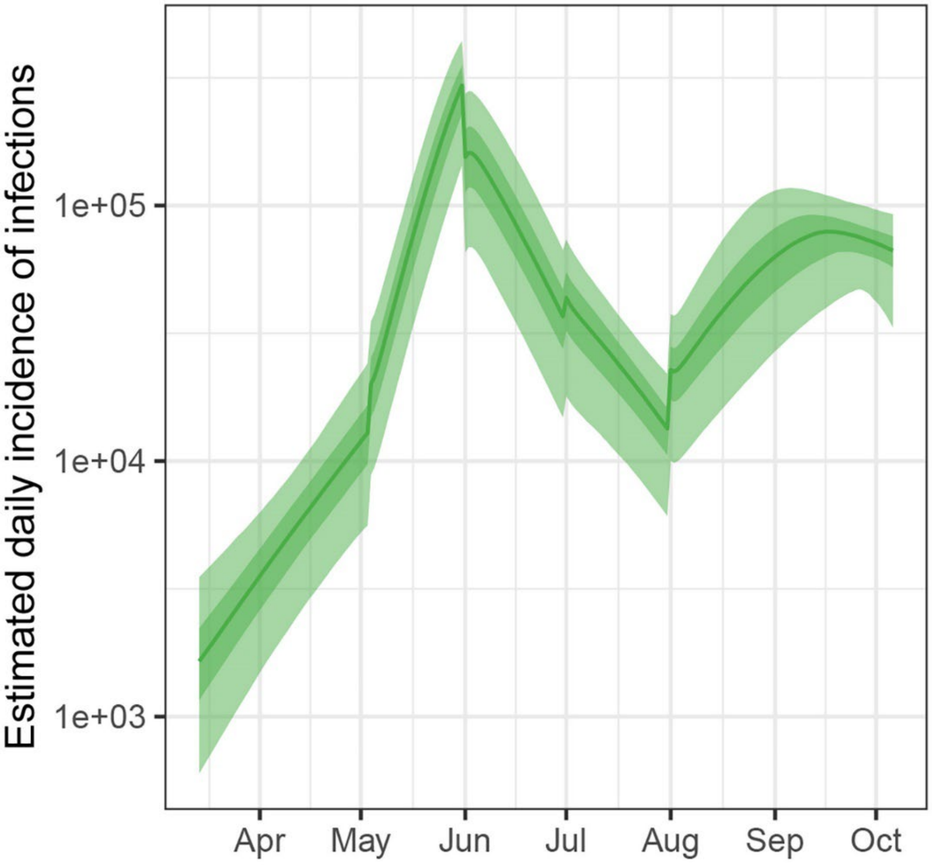

Appendix Figure 5. Reconstructed estimated incidence of infections, with 50% (light green) and 95% (dark green) credible intervals.

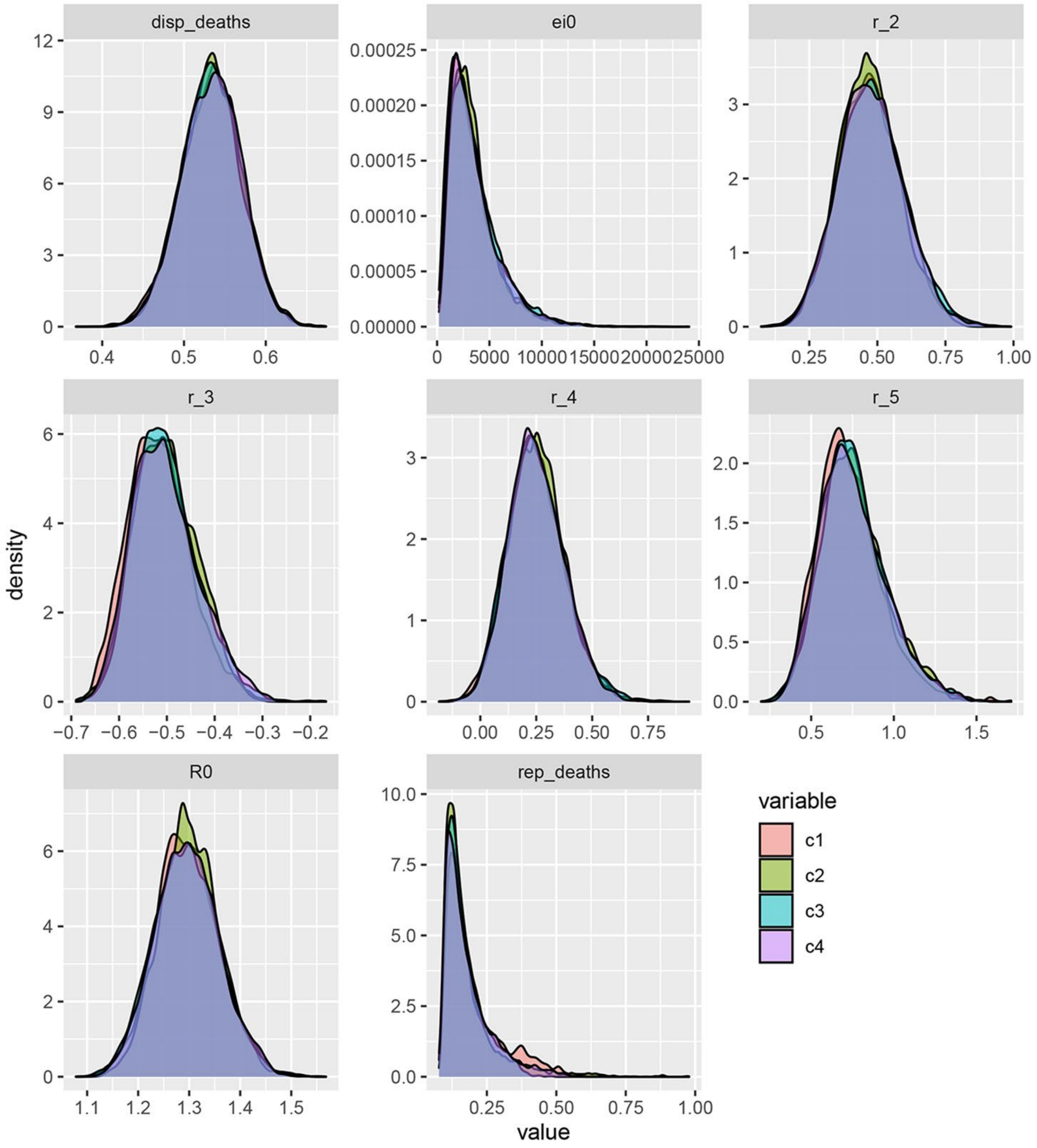

Appendix Figure 6. Posterior densities of estimated parameters for 4 different Markov chain Monte Carlo chains initialised at different starting values.

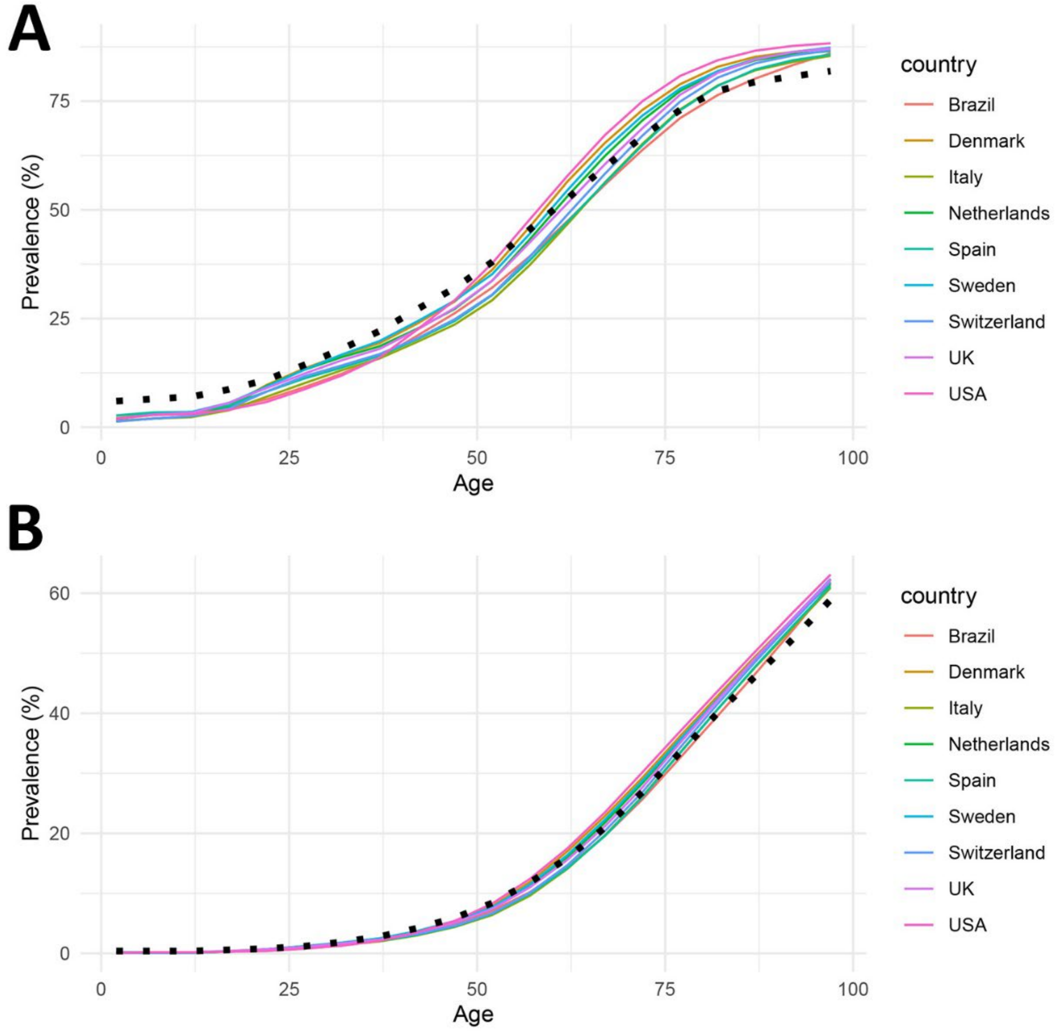

Appendix Figure 7. Prevalence of A) ≥1 or B) ≥2 concurrent conditions for severe COVID-19 by age for India (black dotted line) and the 9 countries that informed the age-specific estimates of the infection fatality ratio used in the model (*9*).
